# Supplementary material for: Processing Parameters and Ion Excipients Affect the Physicochemical Characteristics of the Stereocomplex-Formed Polylactide-b-Polyethylene Glycol Nanoparticles and Their Pharmacokinetics
Source: Pharmaceutics. 2022 Mar 4;14(3):568. doi: 10.3390/pharmaceutics14030568 (PMC8950890; doi:10.3390/pharmaceutics14030568)
Supplement: Supplementary file 1 [file pharmaceutics-14-00568-s001.zip › pharmaceutics-1585728-supplementary.pdf]

pharmaceutics

Supplementary Materials: Processing Parameters and Ion Excipients Affect the Physicochemical Characteristics of the Stereocomplex-Formed Polylactide-b-Polyethylene Glycol Nanoparticles and Their Pharmacokinetics

Kohei Ogawa, Hidemasa Katsumi, Yasushi Moroto, Masaki Morishita and Akira Yamamoto

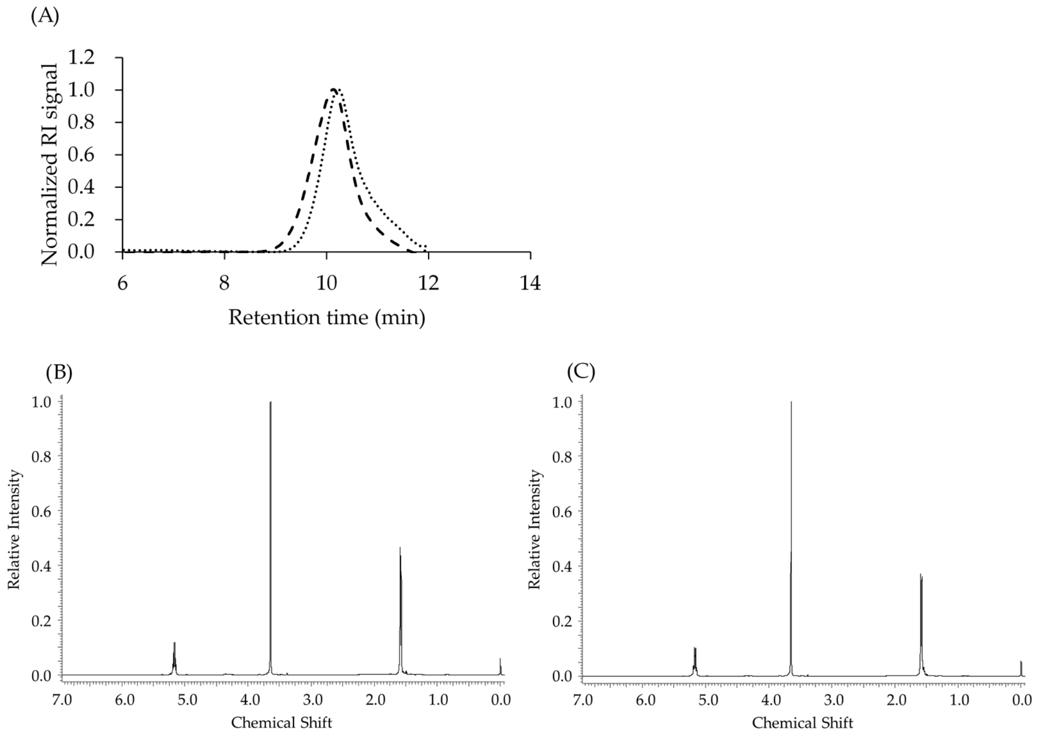

Figure S1. Size-exclusion chromatography–refractive index (SEC–RI) chromatogram and ^{1}H-NMR spectra of PLA-PEG unimers. Panel A indicates SEC–RI chromatograms. The dotted and dashed lines indicate L-lactide base PLA-PEG unimer and D-lactide-based PLA-PEG unimer, respectively. Panels B and C are ^{1}H-NMR plots of L-lactide base PLA-PEG unimer and D-lactide-based PLA-PEG unimer, respectively. The following chemical shifts were assigned: 3.31 ppm, CH3O of methoxy polyethylene glycol; 3.64 ppm, (CH2-CH2-O)$_n$ of methoxy-polyethylene glycol; 5.18 ppm, CH of polylactide; and 1.60 ppm, CH3 of polylactide.

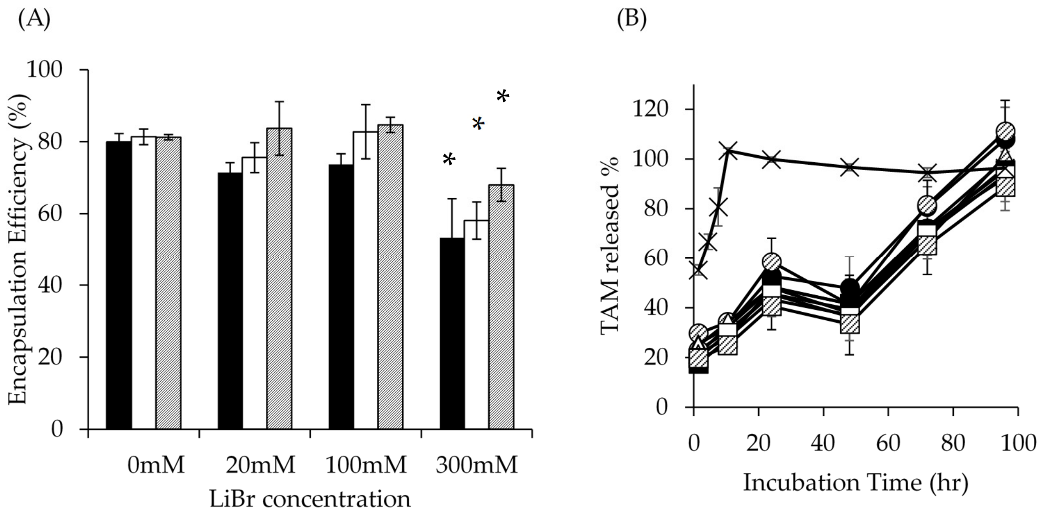

Figure S2. Encapsulation efficiency (EE) and release profile of tamoxifen (TAM) from SC-PEG nanoparticles (NPs). Panel A shows EE quantified by conventional method. Filled, open, and hatched bars indicate flow rates of 8 mL/min, 3 mL/min, and 1 mL/min, respectively. Results are expressed as the mean ± standard deviation of three samples. The experiments were independently performed three times; **p* < 0.05. Panel B shows release profile of TAM from NPs prepared by different process conditions. Closed, open, and hatched symbols indicate flow rates of 8 mL/min, 3 mL/min, and 1 mL/min, respectively. Circle, triangle, and square symbols indicate LiBr concentrations of 0 mM, 20 mM, and 100 mM, respectively. Results are expressed as the mean ± standard deviation of three samples. The experiments were independently performed three times.

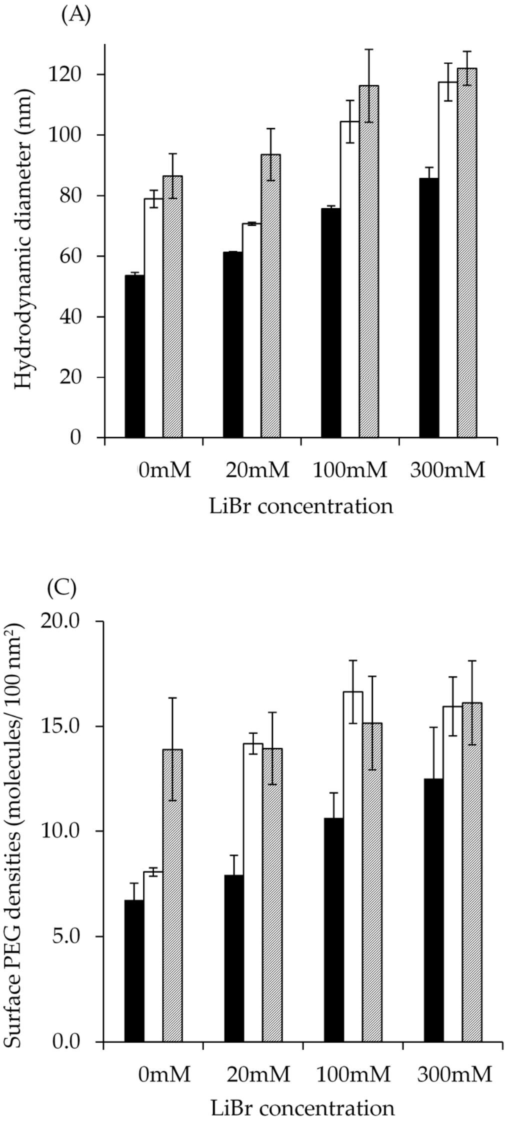

Figure S3. Hydrodynamic diameters (Dh), polydispersity index (PdINP), surface PEG densities, and surface PEG contents of SC-PEG nanoparticles (NPs) prepared by different process conditions. Panels (**A**), (**B**), (**C**), and (**D**) show Dh, PdINP, surface PEG densities, and surface PEG contents of SC-PEG NPs, respectively. Filled, open, and hatched bars indicate flow rates of 8 mL/min, 3 mL/min, and 1 mL/min, respectively. Results are expressed as the mean ± standard deviation of three samples. The experiments were independently performed three times.

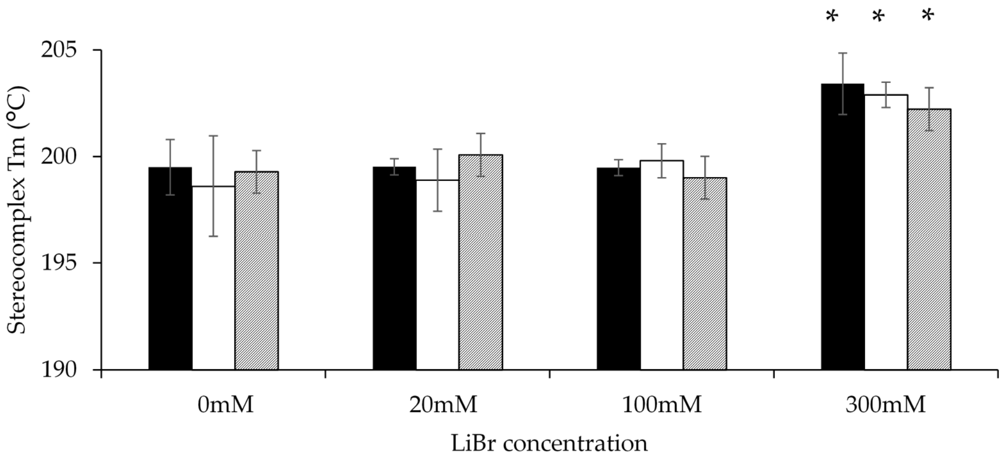

Figure S4. Melting temperature (Tm) of PLA domain in lyophilized SC-PEG nanoparticles (NPs) prepared by different processing parameters. Melting temperature of PLA domain of lyophilized SC-PEG NPs is presented. Filled, open, and hatched bars indicate flow rates of 8 mL/min, 3 mL/min, and 1 mL/min, respectively. Results are expressed as the mean ± standard deviation of three samples. The experiments were independently performed three times; $^*p < 0.05$.

Table S1. Relative molecular weight and polydispersity of SC-PEG unimer in DMF containing LiBr .

LiBr concentration	0 mM	20 mM	100 mM	300 mM
Rel. M_p (g/mole)*	9636	8916	14189	22610
PdI_{uni}	**	1.86	1.11	3.52

* Relative molecular weight of peak top. ** PdI_{uni} cannot be calculated.
